# Calcareous Degeneration of the Hypophysis or Pituitary Body

**Published:** 1890-12

**Authors:** 


### CALCAREOUS DEGENERATION OF THE HYPOPHYSIS OR PITUITARY BODY.

Specimen shown before the Pathological Society by William C. Krauss, M. D.

Name, Theodore Oliver; age, forty-eight years; occupation, tanner; constitution, strong, robust; gave no history of syphilis or tuberculosis, and considered himself a healthy man. In 1880 he passed through some disease of the kidneys, the nature of which could not be ascertained, and supposed himself fully recovered. In July, 1888, he began to complain of pain in the back and base of the head, but still continued his work as cartman. The headaches becoming more severe,—"terrible," as he expressed them,— with attacks of dizziness, neuralgias, and sudden failing of the eyesight, he consulted Dr. Hubbell, of this city, whose examination I here append :

Vision of both eyes impaired, but that of the left eye most. Vision of the right eye equalled No. 12 D of Snellin's test types at five meters, or V=5-12. With a +0.75 D glass he could read a few letters of No. 5 (5-5 partly). With the left eye he could read No. 24 at five meters (L, V=5-24), and glasses aid did not make any improvement.

Both visual fields were contracted, especially at their outer parts (temporarily). The visual field of the right eye was almost entirely lost in its outer half, while that of the left eye was only partially lost. The patient thought he was nearly blind in the right eye, and yet this was the eye in which the vision was much the better. With +2.5 D glasses, he was able to read No. 1 Jaeger's test types at eight inches.

Ophthalmoscopic examination showed the margins of the optic discs somewhat indistinct and fuzzy, while the surfaces of the discs were markedly pale, especially at their outer halves. The vessels were diminished in size, but otherwise normal. The rest of the fundus was in a normal condition. The appearances of the discs were those of a receding optic neuritis and resulting atrophy.

The double optic neuritis, passing into atrophy of the optic nerves, taken in consideration with the dizziness and excruciating headaches, led me to diagnose a tumor of the brain. The bilateral temporal hemianopsia, partially apparent on the left side and well defined on the right side, suggested its location at the base of the brain, and involving the optic tracts or nerves near the chiasma.

The pain soon spread over the whole head, and he suffered much from a sense of fullness and dizziness. About February 1, 1890, the pain became so severe that it was necessary to inject, hypodermically, one grain of sulph. morph. three or four times daily.

August 1, 1890, the pain again became severe, and continued so until August 4th, when he became comatose, and remainedso until his death, the following day.

The brain, as received, was in an advanced state of decomposition, but by immersing it immediately in absolute alcohol, enough of the base was preserved to show the tumor and its surroundings.

Macroscopically the attention is directed at once to the marked increase in size of the pituitary body. Situated normally in the sella turcica, it rarely ever exceeds in size a bean or large pea. At the base of the brain it is found directly caudad of the optic chiasma, and forms the cephalic boundary of the trigonum intercrurale. In this case, it attained the size of a large marble, producing pressure upon the chiasma cephalad, and caudad—it was partially planted in the space between the crura cerebri. On cutting the periphery a gritty, rasping sound was heard, and on cutting deeper the scalpel came in contact with a hard stony substance. The cut surfaces were chalky white, with many glistening points, and crumpled into minute particles.

On examining under the microscope, minute crystals could be seen throughout the field, while the larger particles appeared dark by transmitted light, white and glistening by reflected light.

On adding a drop of strong nitric acid under the cover-glass, these calcareous masses (carbonate of calcium, phosphate of calcium,) dissolved, and the evolution of carbonic acid gas could be distinctly seen under the microscope. After a short lapse of time the field was full of bubbles of gas, some larger and some smaller, positive evidence of the presence of the carbonate of calcium.

For the specimen and symptoms I am indebted to Dr. Hubbell of this city, and to Dr. Witter of Wellsville.

